# Improving Management of Portal Hypertension: The Potential Benefit of Non-Etiological Therapies in Cirrhosis

**DOI:** 10.3390/jcm12030934

**Published:** 2023-01-25

**Authors:** Niccolò Bitto, Gabriele Ghigliazza, Stanislao Lavorato, Camilla Caputo, Vincenzo La Mura

**Affiliations:** 1 Fondazione IRCCS Ca’ Granda Ospedale Maggiore Policlinico, Angelo Bianchi Bonomi Hemophilia and Thrombosis Center, 20122 Milan, Italy; 2 Fondazione IRCCS Ca’ Granda Ospedale Maggiore Policlinico, Division of Sub-Intensive Care Medicine, 20122 Milan, Italy; 3 Department of Pathophysiology and Transplantation, University of Milan, 20122 Milan, Italy

**Keywords:** cirrhosis, portal hypertension, non etiological therapies, albumin, anticoagulation, statins, rifaximin

## Abstract

Portal hypertension is the consequence of cirrhosis and results from increased sinusoidal vascular resistance and hepatic blood inflow. Etiological therapies represent the first intervention to prevent a significant increase in portal pressure due to chronic liver damage. However, other superimposed pathophysiological drivers may worsen liver disease, including inflammation, bacterial translocation, endothelial dysfunction, and hyperactivation of hemostasis. These mechanisms can be targeted by a specific class of drugs already used in clinical practice. Albumin, rifaximin, statins, aspirin, and anticoagulants have been tested in cirrhosis and were a topic of discussion in the last Baveno consensus as non-etiological therapies. Based on the pathogenesis of portal hypertension in cirrhosis, our review summarizes the main mechanisms targeted by these drugs as well as the clinical evidence that considers them a valid complementary option to manage patients with cirrhosis and portal hypertension.

## 1. Introduction

Portal hypertension (PH) is a complication of cirrhosis and represents the primary driver of hepatic decompensation. Structural changes of the liver, namely fibrosis, nodularization of parenchyma (mechanical component), and the development of sinusoidal endothelial dysfunction (dynamic component), cause the initial increase of hepatic resistance to portal blood flow and portal hypertension [1]. The secondary development of splanchnic arterial vasodilation triggers a series of systemic cardio-vascular changes that ultimately foster a hyperkinetic syndrome characterized by high cardiac output, low peripheral resistance, and fluid retention [2,3]. In the presence of high hepatic resistance, the overload of blood volume that reaches the liver because of these systemic hemodynamic changes further increases portal pressure [4]. At this step, patients suffer from so-called clinically significant portal hypertension (CSPH) since the risk of developing the first decompensation parallels the degree of portal pressure [5,6]. Ascites and variceal bleeding are the most frequently observed complications in clinical practice. Etiologic therapies are mandatory to avoid the development of CSPH and passage from a compensated to a decompensated disease, particularly in the early stages [7]. Indeed, the removal of HCV infection, the suppression of HBV replication, the withdrawal of alcohol, the activation of an efficacious weight loss program, and the control of diabetes and dyslipidemia can reduce chronic liver damage, portal pressure, and, ultimately, the clinical risk of decompensation [8,9,10,11]. If this etiological approach is not sufficient to avoid the development of CSPH, non-selective beta-blockers (NSBB) represent the most efficacious strategy to target the hyperkinetic syndrome and reduce portal pressure with a consistent improvement of the clinical outcome [12].

Unfortunately, even after removing the etiologic factors, some patients can remain at a residual risk of PH-related complications [7,13]. This can be the consequence of advanced liver damage at the time of successful etiological therapy, as shown by real-life data in the setting of HCV infection, but it can also be the consequence of other superimposed pathogenic drivers that can independently act on cirrhotic livers and push for a further increase of portal pressure and liver damage [14]. The most credited mechanisms to explain this evolution are inflammation, bacterial translocation, and inappropriate activation of hemostasis [15]. Patients with refractory ascites, one of the most advanced clinical stages of cirrhosis, have high levels of c-reactive protein, which is a marker of systemic inflammation [16]. Similarly, patients with decompensated cirrhosis present circulatory markers of bacterial translocations such as LPS-binding protein, circulatory bacterial DNA fragments, and high levels of TNF-alpha. Several observational studies have demonstrated that the composition of gut microbiota may influence the outcome of cirrhosis [17,18,19,20]. Furthermore, despite the reduction of platelet count and the prolongation of the prothrombin time, patients with cirrhosis present high levels of factor VIII and low levels of protein C, which may support a procoagulant imbalance associated with severe prognosis in cirrhosis [21,22,23]. Moreover, von Willebrand factor, a marker of endothelial dysfunction/activation participating to hemostasis, is elevated along with the severity of the liver disease, correlates with portal pressure, and endotoxemia, and has been consistently associated with prognosis, suggesting that endothelial damage and over-activation of hemostasis could be pathogenic mediators of liver damage [17,21,24,25]. Importantly, all of these mechanisms can be targets of therapy in cirrhosis. Albumin, rifaximin, statins, aspirin, and anticoagulants are drugs that have received considerable attention from hepatologists in recent years, and their use in the management of patients with portal hypertension was discussed in the last Baveno consensus as non-etiological therapies [5].

**Figure 1:**
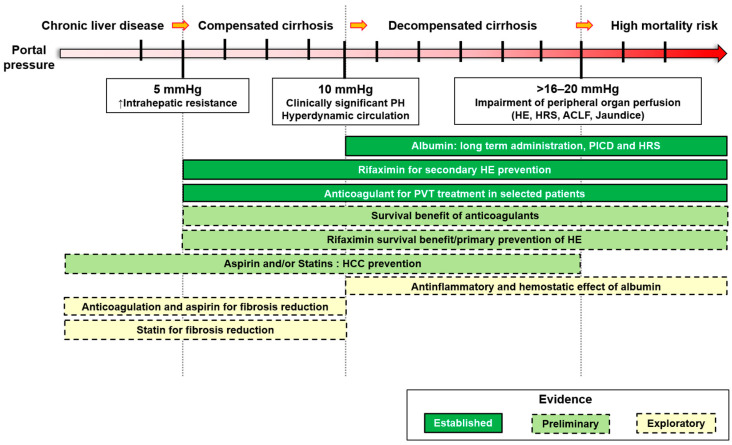
Non-etiological therapies, in relation to portal hypertension level and grade of evidence. PICD, paracentesis induced circulatory dysfunction; HE, hepatic encephalopathy; HRS, hepatorenal syndrome; ACLF, acute on chronic liver failure; HCC, hepatocellular carcinoma; PVT, portal vein thrombosis.

This review describes the main pharmacological strategies helpful in managing cirrhosis based on these pathophysiological targets over an etiological approach. Each drug will be discussed in detail, including targeted mechanisms, pharmacological properties, and the clinical evidence available for its use in the different stages of cirrhosis (Figure 1).

## 2. Albumin

Albumin is a globular, water-soluble, 67 kDa protein synthesized by hepatocytes (10–15 g/die). Its concentration in human blood ranges from 3.5–5 g/dL and accounts for about half of serum proteins [26,27,28]. Its metabolism is guaranteed by the continuous uptake of oxidized albumin by hepatocytes through a pH-mediated mechanism of endocytosis, which provides for stable, non-oxidized molecules [29].

Albumin is fundamental for homeostasis, with scavenging, immunomodulant, and antioxidant properties. Thanks to its stable but flexible structure, it is also involved in solubilization and transport of other endogenous and exogenous molecules [30]. Among these, many drugs, hormones, nitric oxide, endotoxins, inflammation mediators, bilirubin, and bile acids are the most important and account for albumin’s biological properties. Albumin inhibits TNF α-induced upregulation of vascular cell adhesion molecule-1 (VCAM-1) and NF-kB activation, thus enhancing endothelium protection against inflammation and oxidative stress [30,31].

Albumin is one of the mainstays of treatment in some significant cirrhosis-related complications: paracentesis induced circulatory dysfunction (PICD), acute kidney injury-hepatorenal syndrome (AKI-HRS), and spontaneous bacterial peritonitis (SBP). PICD is a complication that can occur secondary to the detrimental hemodynamic effects of large-volume paracentesis [32,33]. It is characterized by prerenal acute kidney injury and further reactivation of the renal–angiotensin system with worsening hyponatremia and volume congestion, and it has a potential life-threatening effect on prognosis. International guidelines recommend albumin supplementation when fluid removed by paracentesis is more than 5 L (8 g of albumin/liter of removed ascites) to prevent this harmful complication [34]. Several studies have also evaluated the impact of chronic use of albumin in patients with ascites [35,36]. Promising data derived from the ANSWER study, a randomized clinical trial in cirrhotic patients with grade 2 or 3 ascites, which tested the efficacy of chronic albumin supplementation (40 g twice weekly for 2 weeks, and then 40 g for up to 18 months) on top of the standard of care. Albumin showed an increased 18-month survival and reduced need of large volume paracentesis over time [35]. These results were confirmed by a subsequent observational study [37], and the Italian Association for the Study of Liver has recommended the chronic use of albumin in non-complicated grade 2 ascites as a treatment option [38]. AKI-HRS is a unique form of kidney injury in cirrhotic patients with ascites that is caused by circulatory and inflammatory dysfunction. Vasodilation and reduced cardiac output account for renal hypoperfusion, whereas inflammation and microcirculatory dysfunction directly damage proximal epithelial tubular cells [39]. International guidelines suggest treating any AKI, in the absence of other overt renal and post-renal causes, with the withdrawal of nephrotoxic drugs (angiotensin-converting-enzyme inhibitors, non-steroidal anti-inflammatory drugs, diuretics) and volume expansion with albumin at the dose of 1 g/kg (with a maximum of 100 g of albumin) over 48 h [40]. The diagnosis of AKI-HRS is made in cases of non-response to this volume expansion, and vasoactive drugs and albumin at the dosage of 20–40 g/day must be initiated to improve renal perfusion and dampen inflammation [40,41]. SBP is the infection of ascitic fluid without any intra-abdominal surgically treatable source, and it is diagnosed by a neutrophil count of more than 250/mm^3^ on the ascitic fluid [42,43]. SBP, like any other infection, may further decompensate cirrhosis by inducing systemic vasodilation and inflammation as well as worsening hyperdynamic circulation [44]. Alongside proper antimicrobial treatment, albumin is administered at a dosage of 1.5 g/kg body weight at diagnosis, followed by 1 g/kg on day three, with the primary clinical benefit of AKI-HRS prevention and mortality reduction [40,45].

Along with these solid clinical indications, albumin treatment has been explored in other clinical conditions. The INFECIR 2-study tested albumin administration in non-SBP acute bacterial infections, without significant effects on in-hospital mortality, which was the primary endpoint of the trial [46]. The ATTIRE study failed to show any benefit from albumin in non-infected patients with hypoalbuminemia hospitalized for acute decompensation [47]. The PRECIOSA study assessed the significant decrease in proinflammatory cytokine and the improvement of hemodynamic state in patients with long-term albumin treatment [48]. Nevertheless, even in the chronic setting, albumin had to be administered at a high dosage (1.5 g/kg/week) to achieve a significant increase in its serum levels and replenish non-oxidized albumin, which is the most biologically active circulating form of the molecule [48,49,50,51].

The number of randomized controlled trials and observational studies published on albumin in the setting of cirrhosis reveals the great interest of hepatologists in this molecule. Indeed, it has pleiotropic effects that cover the traditional control of oncotic pressure as a plasma expander and, more importantly, the modulation of systemic inflammation, oxidative stress, and endothelial function. Indeed, exploratory investigations associated hypoalbuminemia with higher levels of von Willebrand factor, thus implying an additional role on hemostasis [44,52]. Interestingly, in patients with spontaneous bacterial peritonitis, albumin infusion reduced von Willebrand factor, procoagulant factor VIII and endothelial dysfunction [44,53,54]. Hypoalbuminemia also seems to be a better predictor of venous thromboembolism than prothrombin time and platelet count [55]. All these properties are associated with the clinical effect of albumin and confirm its role as the first-choice plasma expander in patients with cirrhosis. However, despite clear-cut indications for the prevention of PICD, the management of SBP and AKI-HRS, the use of albumin out of these conditions needs further exploration to maximize the high potential of this molecule in daily clinical practice.

## 3. Rifaximin

Rifaximin is an oral non-systemic antibiotic with broad-spectrum microbial activity, due to the inhibition of bacterial RNA polymerase [56]. It is water-insoluble and poorly absorbable, with ideally null systemic bioavailability [57,58]. Rifaximin shows in vivo direct bactericidal activity against Gram-positive, Gram-negative, and aerobic and anaerobic bacteria, making it an efficacious tool to modulate gut microbiota with an impact on bacterial translocation [59]. These pharmacological properties are of potential interest in the management of cirrhosis. Several authors have demonstrated that gut microbiota is pivotal in systemic homeostasis and interacts with the liver in the so-called “gut-liver axis”, which integrates the bidirectional relationship among these two organs with signals from diet, genetic background, and environment [60]. All these issues may influence the evolution of cirrhosis toward decompensation, further decompensation, and acute on chronic liver failure (ACLF), a condition with high short-term mortality characterized by multiple organ dysfunction that are triggered by several precipitating factors, including alcohol abuse and bacterial infections [61,62]. High levels of inflammation seem to predispose individuals to this life-threatening condition, and emerging evidence links such catastrophic inflammatory state to bacterial translocation, which is incremental in the most advanced stages of cirrhosis [63,64,65,66]. Although some differences have been described among different etiologies of chronic liver disease [67,68], gut microbiota alteration represents the common ground to explain several detrimental clinical effects of cirrhosis [69,70,71]. In the latest stages of cirrhosis, concomitant gut motility impairment and bile acid metabolism reduce physiological control of bacterial translocation [20,72], which leads to bacterial overgrowth [70]. For these reasons, several authors have tested the efficacy of rifaximin to modulate the gut microbiota and ultimately reduce the rate of clinical complications in cirrhosis, with promising but not definitive results. An observational study associated rifaximin with the reduction of portal pressure that is due to decreased endotoxin plasma levels [73], whereas other trials in patients with ascites failed to demonstrate any clinical effect [74,75]. A randomized controlled trial comparing propranolol vs propranolol plus rifaximin showed a higher reduction of portal pressure in the latter group [76]. Another study demonstrated that patients receiving rifaximin had a significantly lower risk of variceal bleeding, AKI-HRS, and better survival [77]. Although the number of studies suggesting beneficial effects of rifaximin in cirrhosis is growing [78,79,80,81,82], some authors continue to report negative results [83,84,85]. Today, a formal indication of rifaximin in the management of patients with cirrhosis is restricted to preventing recurrent hepatic encephalopathy (HE) [86]. Rifaximin significantly improves quality of life, HE recurrence, and prognosis [87,88,89]. Moreover, in a recent trial, rifaximin prevented the incidence of overt HE after transjugular intrahepatic portosystemic shunt (TIPS) placement in cirrhosis, indicating that it is also useful in this setting [90]. An expanded use of rifaximin outside of those indications is not recommended, even though the microbiota modulation could also have important immunomodulant and portal hypotensive effects of potential interest in non-encephalopathic patients. A widespread use of rifaximin would also create concerns about antibiotic resistance. Emerging multi-drug resistant organisms (MDRO) represent a worldwide problem, as they are associated with increased mortality, septic shock, longer hospital stays, and intensive care unit admission [91]. Cirrhotic patients are a high-risk population for MDRO-related complications because of their intrinsic frailty, repeated hospitalizations, invasive procedures, and prophylactic exposure to antibiotics [92]. Moreover, MDRO are associated with progression to decompensated cirrhosis and, ultimately, to ACLF [93]. Despite some favorable results on this issue [94,95]. Clostridium difficile outbreaks have been reported [96], with an increasing rate of resistant strains from 8 to 35% [97]. Significantly, rifaximin use was not associated with rifamycin-resistant strains of C. difficile, but further studies are warranted on this safety issue [97]. Collectively, despite promising results, significant caveats for the widespread use of rifaximin exist and restrict its use to only approved indications.

## 4. Statins

Statins are among the most prescribed class of medications worldwide and an increasing number of patients have received statins for primary or secondary prophylaxis of cardiovascular events in the last decades in all developed countries [98]. Several studies have demonstrated that statins significantly reduce cardiovascular risk, which remains one of the most frequent causes of morbidity and mortality worldwide [99].

A well-studied mechanism of statin activity is the inhibition of HMG-CoA reductase, which effectively reduces blood cholesterol levels [100,101]. In addition to lowering cholesterol levels, statins have pleiotropic effects, mainly by interfering with the isoprenylation of proteins, which ultimately controls a series of transcription factors related with inflammation, angiogenesis, fibrosis, and endothelial function and may be beneficial in several chronic inflammatory conditions [102].

For these reasons, statins have been investigated as a potential treatment option in chronic liver diseases. Statins may reduce hepatic steatosis by activating sterol regulatory element-binding proteins (SREBPs), peroxisome proliferator-activated receptor alpha (PPARα), and β-oxidation, even if their beneficial effects in experimental steatosis remain controversial [103]. However, statins decrease inflammatory response through various mechanisms. First, they reduce tumor necrosis factor α (TNFα), interleukins 1-beta (IL1β) and 6 (IL6), and C-reactive protein (CRP) by modulating PPARγ activity [104,105]. Second, statins directly inhibit the expression of major histocompatibility complex class II molecules in CD4+ helper T cells (TH1 cells), leading to a shift toward anti-inflammatory TH2 cell actions [106]. Finally, statins reprogram endothelium phenotype to be anti-inflammatory, thereby modulating Krüppel-like factor 2 (KLF2) [107,108]. These properties may have a crucial effect on liver disease since systemic inflammation is increasingly recognized as the hallmark of cirrhosis progression [109]. Moreover, through the modulation of KLF2, statins impact the dynamic component of portal hypertension by interfering with pathways that regulate the vascular tone controlled by sinusoidal endothelial cells. Indeed, statins increase the bioavailability of nitric oxide by positively regulating the expression of endothelial nitric oxide synthase (eNOS) [110]. This property successfully preserved liver vascular tone in rat models of cirrhosis, ischemia-reperfusion injury, and endotoxemia [110,111,112,113]. Moreover, statins may beneficially impact pathological angiogenesis, but evidence is preliminary and no data exist on macrovascular complications such as shunt/collateral development [114,115,116]. The clinical efficacy of these findings has been tested in patients with portal hypertension, and simvastatin is the most investigated statin in human studies on cirrhosis. Simvastatin decreases portal pressure after meal intake as measured by changes in the hepatic venous pressure gradient (HVPG) [117]. Moreover, it decreases HVPG and improves liver perfusion with complementary effects on those related to non-selective beta-blockers (NSBB) [118]. When tested in a pragmatic double-blind trial on cirrhotic patients who had recovered from variceal bleeding, simvastatin on top of the standard of care for secondary prophylaxis (e.g., NSBB plus variceal band ligation) did not impact the primary endpoint of rebleeding prevention. However, it was associated with a benefit on survival in patients with Child–Pugh class A or B, which was the secondary endpoint of the trial [119]. Retrospective studies also showed a survival benefit in cirrhotic patients who received statin therapy [120,121,122].

Beyond the impact on portal hypertension, statin therapy has been associated with other pleiotropic hepatological effects. First, in different experimental models of chronic liver injury, statins affected the paracrine signaling of hepatocytes on hepatic stellate cells (HSCs), blocking hepatic stellate cell (HSC) activation and fibrogenesis [123]. Second, statins were associated with reduced progression to advanced fibrosis and cirrhosis in registry and biopsy-proven studies [124,125]. Moreover, several observational studies hypothesized a chemopreventive effect on HCC development [126,127]. Recently, in a rat model of endotoxemia, simvastatin has demonstrated intrahepatic anti-thrombotic properties able to counteract sepsis-associated coagulopathy and liver damage [128]. This is in line with the potential anti-thrombotic effects of statins, where anti-inflammatory properties are targeted to the endothelium [129,130]. These observations have increased hepatologists’ interest in the prescription of statins to ameliorate the management of patients with cirrhosis. However, some safety issues remain that globally limit the use of these drugs in cirrhosis. The main side effect of statins is muscular toxicity, while drug-induced liver injury related to statins is infrequent (<2 cases/1,000,000 patient-years) and likely idiosyncratic [131,132,133]. Despite no serious adverse events related to statins were reported in two randomized controlled trials that evaluated the effect of simvastatin on portal pressure [118,134] in another large trial of statins in cirrhosis, 2/69 patients treated with simvastatin at a 40 mg/day dosage developed rhabdomyolysis [119]. Patients with advanced stages of cirrhosis could theoretically be at increased risk of adverse effects because they are more prone to the consequences of drug-induced impairment of CYP3A4 metabolism in the liver [135]. Therefore, in patients with decompensated cirrhosis, simvastatin may elicit rhabdomyolysis and hepatotoxicity at a 40 mg daily dose [136] while, in normal circumstances, such adverse effects are rare [137]. The reasons might be related to the dose of statins, and genetic predisposition (e.g., SCLO1B1 polymorphism). Moreover, the alcoholic etiology of liver disease could be an additional and non-negligible predisposing factor of drug toxicity. Generally, the benefits of statins outweigh their potential hepatotoxic risks; therefore, the continuation or initiation of therapy is recommended for the management of the cardiovascular risk in cirrhosis, along with the close follow up for muscle and liver toxicity [5]. Future interventional studies should clarify whether statins may have a primary hepatological indication as a disease-modifying drug for cirrhosis and portal hypertension.

## 5. Aspirin

Aspirin is a commonly prescribed drug that exerts its anti-inflammatory activity through irreversible cyclooxygenase (COX) inhibition, leading to reduced prostaglandins E and I production [138]. Moreover, acetylation of platelet COX by aspirin blocks the formation of thromboxane (TXA), which is a mediator of vasoconstriction and platelet aggregation [139].

In recent years, numerous pre-clinical and clinical studies have demonstrated the potential liver-specific effects of aspirin, particularly a protective anti-fibrogenic and antiproliferative activity that was demonstrated in cellular and animal studies, with a potential clinical impact on the evolution of hepatic fibrosis and reduction of the risk of HCC [140].

When the liver is damaged, the production of profibrotic cytokines such as TGF-β1 (transforming growth factor-β1) and TNF-α (tumor necrosis factor-α) ensues. TGF-β1 and TNF-α, in turn, activate hepatic stellate cells (HSCs), which start collagen production. This ultimately causes extracellular matrix (ECM) remodeling and liver fibrosis [141,142,143]. In pre-clinical models, aspirin has demonstrated a potential modulation of some critical players of liver fibrosis [25]. In a rat model of fibrosis induced by thioacetamide (TAA), aspirin downregulated TGF-β1 signaling [144]. In particular, it reduced the expression of p-Smad2 and p-Smad3, which participate in the transcription of liver fibrosis-related genes such as fibronectin, α-SMA and collagen-I [144]. Moreover, higher aspirin doses were associated with reduced TAA-induced liver fibrosis [145]. Platelets and platelet-derived growth factor-β (PDGF-β) are other potential targets of aspirin. PDGF-β boosts the activation of quiescent HSCs into myofibroblasts and activated platelets release TGF-β and chemokine ligand 4 [146]. In MDR2^−/−^ mice, a model of chronic biliary damage, platelet clusters were spotted in the hepatic endothelium of fibrotic livers and recognized as a source of PDGF-β [147]. Administration of low dose of aspirin to MDR2^−/−^ mice reduced fibrosis progression over one year. Clinical observations on the impact of aspirin on fibrosis are substantially limited to patients suffering from a chronic metabolic disease of the liver. In a cross-sectional study, regular aspirin administration was associated with a 38% risk reduction of being diagnosed with NAFLD, particularly in male and older patients [148]. Moreover, in a cohort of 1857 individuals with suspected liver disease, aspirin use was associated with lower non-invasive fibrosis indexes (e.g., FIB4, APRI, Forns and NFS) [149]. These findings were limited by the retrospective nature of the studies, absence of data on non-steroidal anti-inflammatory drugs (NSAIDs), and the diagnosis of steatosis made only by ultrasonography. Notwithstanding, Simon et al. recently demonstrated that, in 361 adults with biopsy-proven NAFLD, patients on regular aspirin therapy had a lower risk of histological fibrosis and steatohepatitis at baseline and a reduction of both a worsening rate of liver stiffness and non-invasive tests of fibrosis (FIB4, APRI, NFS) [150].

The decrease of inflammation and fibrosis induced by aspirin makes an indirect benefit on the main complications related to chronic liver damage plausible. This idea is supported by exploratory experimental and clinical data on the effect on HCC and the development of portal hypertension. Aspirin may exert a chemopreventive effect on the liver through various mechanisms. It targets NFkB proinflammatory signaling and decreases glucose uptake of hepatoma cells through the downregulation of glucose transporter 1 (GLUT1), leading to the inhibition of proliferation [151,152]. Aspirin may also reduce in vivo and in vitro cell proliferation and sensitize to anti-neoplastic agents [153,154,155]. Since platelets can adhere to tumor cells, the anti-platelets effect may curb the pro-metastatic tumor environment, thus limiting immune clearance and producing pro-angiogenic and growth factors, such as TXA2 [156]. Moreover, beyond the effect on structural changes in the liver, aspirin can potentially revert the dynamic changes that cause intrahepatic vasoconstriction and portal hypertension. Indeed, COX-1/TXA2 have critical roles in regulating vasoconstriction of liver sinusoids, and endothelial dysfunction in CCl_4_ cirrhotic rat liver has been associated with increased TXA2 [157,158]. Non-specific and specific COX-1 inhibition corrected endothelial dysfunction in this animal model, demonstrating the potential pathophysiological effect of COX-1 inhibition on portal hypertension [158].

Clinical studies exploring the potential role of aspirin in preventing advanced liver complications are substantially limited to HCC risk reduction. In a population-based study in the US that included 1,084,133 individuals, HCC was diagnosed in 676 patients, and aspirin use was associated with a 32% reduction of risk. The protective effect of aspirin was stronger for lower dosages and longer duration of daily use (>5 years). The association was also confirmed in retrospective large studies of high-risk populations (viral hepatitis/cirrhosis) [159,160,161,162,163,164,165,166]. A nationwide study from Swedish registries explored the impact of the new use of low-dose aspirin (≤160 mg) on the incidence of HCC in patients with confirmed chronic hepatitis B or hepatitis C [166]. Aspirin was started at least 180 days after the diagnosis of hepatitis and was associated with 31% lower risk of HCC than non-users. In this experience, the inverse relationship between aspirin and the risk of HCC also appeared to be time dependent. In addition, the adjusted risk of liver-related death was 27% lower for aspirin users without increased gastrointestinal bleeding events. These effects seem to be related only to aspirin, without benefit for the other NSAIDs [167]. These data led to multiple evaluations in systematic reviews and metanalyses, confirming the effect of aspirin on the chemoprevention of HCC [167,168,169,170]. However, this beneficial effect seems less evident in advanced stages of cirrhosis [160], and another meta-analysis reports conflicting results on this issue [171]. Altogether, these data advocate for higher quality evidence from prospective and interventional studies aimed to test aspirin for the primary endpoint of HCC prevention in high-risk populations, with proper stratification for disease severity, since safety issues are striking different in cirrhosis [172,173].

The clinical role of aspirin in portal hypertension has been limited by safety concerns that are due to the thrombocytopenia frequently observed in patients with cirrhosis [173,174]. Notwithstanding, retrospective evidence on aspirin use for cardiovascular indication in patients with cirrhosis showed no significant increase in major bleedings [175,176,177,178,179]. Moreover, a recent multicenter retrospective study including 587 participants evaluated the impact of aspirin prescribed after TIPS placement. In this experience, aspirin significantly modified transplant-free survival at 12 months after TIPS in patients with refractory ascites, while no remarkable effects were observed in the group receiving TIPS for refractory variceal bleeding [180].

These findings collectively led the last Baveno consensus to indicate that aspirin could be prescribed for cardiovascular reasons in cirrhosis, since the potential beneficial effects of aspirin as summarized above suggest that cirrhosis should not be considered an absolute contraindication for aspirin’s use in clinical practice [5]. However, several questions remain before aspirin can be indicated as an appropriate disease-modifying drug in cirrhosis. The clinical benefits of aspirin in cirrhosis are based on observational studies, with significant heterogeneity in dose, duration, and follow-up time. Furthermore, patients taking aspirin for cardiovascular reasons are likely to assume other concurrent medications that could potentially affect liver disease (e.g., metformin, statins, angiotensin-convertor inhibitors). In addition, the effect on portal hypertension has not been adequately addressed in human studies and safety concerns can be suggested in decompensated cirrhosis since COX inhibition can lead to kidney damage in such a fragile group of patients. The increasing epidemiological prevalence of liver metabolic disease and the potential increase of cirrhotic patients who need an anti-platelet drug to prevent cardiovascular events [157,158,159,160,161] makes it necessary to explore the real benefit risk-ratio of aspirin in advanced chronic liver disease.

## 6. Anticoagulation

The use of anticoagulants in cirrhosis has been limited for a many years by the belief that patients with advanced chronic liver disease were at a disproportionate risk of bleeding [181]. Indeed, cirrhosis was considered the prototype of acquired hemorrhagic diseases. While laboratory tests suggest hemostatic alterations in bleeding diathesis, there is now strong experimental and clinical evidence that coagulation is rebalanced in chronic liver disease [182]. Moreover, despite the characteristic thrombocytopenia of chronic hepatitis and cirrhosis, in vitro tests suggest that these platelets adequately adhere to collagen [183], and thus do not directly contribute to the bleeding risk, at least for values >50,000/mm^3^ [184]. Coagulation in cirrhosis is currently viewed as a precarious balance between procoagulant and anticoagulant factors, exposing patients to the risk of bleeding episodes and thrombotic events [185]. Portal vein thrombosis (PVT) is a frequent complication of cirrhosis and is associated with the advanced stages of the disease, paralleling the slowing of portal flow and the unbalanced hemostasis [186]. Management of PVT consists of anticoagulation with low molecular weight heparin (LMWH) and vitamin K antagonists (VKA) [187]. As for chronic PVT, the main challenge is analyzing the risk of hemorrhage compared to the advantage of facilitating recanalization and avoiding thrombus extension. While acute treatment is mandatory in patients with ischemic symptoms, the net clinical benefit of chronic therapy in asymptomatic patients should be individually assessed. The inclusion of the patient in the transplant list is a fundamental factor for the decision, as the vascular anatomy is critical for the technical feasibility of the anastomosis of liver graft with a proven impact on survival post liver transplantation [188]. However, room for clinical benefit beyond the anatomical viability of liver transplantation should be considered. In a meta-analysis of eight studies including 353 patients with cirrhosis and PVT, patients treated with anticoagulants (LMWH or warfarin) had higher recanalization rates than untreated patients (71 vs. 42 percent) and reduced risk of major bleeding [189]. Moreover, anticoagulation was not associated with excessive bleeding events in the context of endoscopic band ligation [190]. It should be kept in mind that upper-gastrointestinal bleeding during VKA in cirrhosis is not due to the anticoagulant treatment but is mainly related to portal hypertension. This was demonstrated in a retrospective study involving patients with and without cirrhosis, through comparisons between three cohorts: patients with cirrhosis and PVT receiving VKA, patients with cirrhosis not receiving VKA, and patients without cirrhosis who received VKA to prevent venous thromboembolism [191]. In this experience, the rate of upper-GI bleeding in the PVT cohort was comparable with that of patients with cirrhosis without PVT who were not exposed to anticoagulation. Nevertheless, complete recanalization of PVT during therapy with VKA reduced portal hypertension-related events and improved transplantation-free survival, conferring a benefit on the evolution of cirrhosis [191]. These data were confirmed by independent observational studies [192,193] and a subsequent meta-analysis [194]. Moreover, in patients with PVT who required TIPS placement, anticoagulant therapy was associated with an increased recanalization rate, suggesting a synergistic effect [195,196].

Beyond macrovascular complications, a growing body of evidence demonstrates that a prothrombotic state may accelerate the development of fibrosis and cirrhosis through microvascular ischemia [25,197]. Endothelial damage and microvascular thrombosis may lead to hepatocyte ischemia and loss of functional hepatocytes, as postulated by the so-called parenchymal extinction hypothesis [198,199]. Furthermore, the prognostic role of an imbalance of prothrombotic factors has been associated by independent reports with decompensations and higher mortality [22,200,201,202]. Several studies on different murine models of chronic liver disease supported the evidence of anti-hemostatic interventions to prevent liver damage and fibrosis [145,203,204,205,206,207,208,209]. On the molecular level, thrombin may elicit HSCs activation via protease-activated receptors (PARs), and anticoagulants may curb this profibrotic signaling [25,210,211,212,213]. The most robust clinical evidence on the disease-modifying role of anticoagulants on cirrhosis comes from a seminal non-blinded randomized trial, in which 70 cirrhotic patients were randomly assigned to receive enoxaparin (4000 IU/day for 48 weeks) or no treatment, with the primary objective of preventing PVT [214]. At 48 weeks, none of the patients in the enoxaparin group had developed PVT, compared with 16.6% of controls. Moreover, liver decompensation was less frequent among patients given enoxaparin (11.7%) than among controls (59.4%), with increased survival. Collectively, evidence is in favor of maintaining anticoagulation in cirrhotic patients when there is an approved indication for its use since it may reduce liver-related outcomes in patients with and without PVT and improve overall survival [187,215]. Investigations on the use of direct oral anticoagulants (DOACs) in this context are currently limited in cirrhosis, despite preliminary reports showing similar efficacy and reduced bleeding events, at least when DOAC are indicated for cardiovascular reasons [216,217,218,219,220]. Interestingly, a recent report on cirrhotic patients with atrial fibrillation associated DOACs with reduced all-cause mortality, reduced bleeding, and a tendency to reduce hepatic decompensations [221]. As commented above regarding aspirin, efforts should be made to avoid under-utilization of anti-coagulation in cirrhosis, while adequately designed prospective and interventional studies are warranted to test anti-coagulant interventions with the primary endpoint of overall liver-related survival, prevention of decompensation, and safety.

## 7. Conclusions

In recent years, a significant number of studies focused on the pathophysiology of cirrhosis and portal hypertension has allowed for the detection of a series of new pathophysiological targets which have high potential to modify the clinical course of advanced chronic liver disease on top of the traditional etiologic approach (Table 1). Albumin and rifaximin are drugs that already have a formal indication to treat several complications of cirrhosis and portal hypertension. However, there is room to better characterize the subset of patients who could benefit most from the wide range of properties disclosed by these molecules against inflammation, oxidative stress, and bacterial translocation. Recently, special attention has been directed toward statins. Simvastatin has been the most studied statin in humans. We know today that it reduces portal pressure in patients treated with NSBB and, potentially, may ameliorate survival in patients who require rebleeding prophylaxis. These important effects are based on the ability of the drug to ameliorate endothelial function over its lipid-lowering effect. This notwithstanding, safety concerns raised by the most recent reports are a crucial cautionary note before giving final approval on a primitive hepatological indication to simvastatin in clinical practice. Anticoagulants and aspirin may also provide some beneficial hepatological effects. The hyperactivation of hemostasis in cirrhosis is traditionally associated with PVT; however, several authors have suggested that targeting hemostasis could reduce liver damage at the level of microcirculation as well as endothelial dysfunction and inflammation. Unfortunately, the lack of randomized controlled trials against hard clinical endpoints is a significant limitation to translating these data into clinical practice.

## Figures and Tables

**Table 1:** Summary of established and potential non-etiological therapies in cirrhosis and portal hypertension and future research agenda in this area.

Drug	Main Established and Potential Non-Etiological Effects	Research Agenda
Albumin	➢ Prevention of PICD after LVP [34] ➢ Hepatorenal syndrome [40,41] ➢ Increased survival in chronic supplementations [35] ➢ Anti-inflammatory properties [44,53,54]	➢ Personalized care to maximize the beneficial effects in patients with infection ➢ Long term administration should be compared with alternative strategies for affordability
Rifaximin	➢ Secondary prevention of HE [86] ➢ Primary prevention of HE after TIPS [90] ➢ Gut-liver axis modulation, reduction of cirrhosis complications [78,79,80,81,82]	➢ RCT against hard-clinical endpoints: decompensation, survival ➢ Safety data on emergent MDRO
Statins	➢ Reduction of portal pressure [118] ➢ Mortality reduction [119,120,121,122] ➢ Reduction of endothelial dysfunction [110,111,112,113] ➢ Fibrosis reduction in chronic hepatitis [124,125] ➢ Reduction in HCC incidence [126,127]	➢ RCT against hard-clinical endpoints: HCC, survival ➢ Expanding data on safety in advanced stages of cirrhosis
Aspirin	➢ Fibrosis reduction in chronic hepatitis [144,145,149,150] ➢ Reduction in HCC incidence [159,160,161,162,163,164,165,166] ➢ Potential reduction of PH [158]	➢ Addressing safety issues in advanced cirrhosis, (thrombocytopenia and kidney failure the main concerns) ➢ RCT against hard-clinical endpoints: first decompensation, HCC, survival
Anti-coagulants	➢ PVT recanalization in acute and chronic setting in selected patients [187] ➢ Survival benefit in PVT recanalization [194] [189] ➢ Survival benefit in observational studies and in a single RCT in decompensated cirrhosis [214] ➢ Emerging promising data on DOACs in PVT and cardiovascular indications in cirrhosis [216,217,218,219,220,221]	➢ RCT in chronic PVT ➢ RCT against hard-clinical endpoints: decompensation, HCC, survival ➢ Safety and efficacy data on DOAC

PICD, post paracentesis circulatory dysfunction; LVP, large volume paracentesis; HE, hepatic encephalopathy; TIPS: transjugular intrahepatic portosystemic shunt; HCC, hepatocellular carcinoma; PH, portal hypertension; PVT, portal vein thrombosis; DOAC, direct oral anticoagulant; RCT, randomized controlled trial.

## Data Availability

Not applicable.
